# Toxic epidermal necrolysis-like subacute cutaneous lupus erythematosus following pembrolizumab therapy

**DOI:** 10.1016/j.jdcr.2025.06.034

**Published:** 2025-07-07

**Authors:** Emma Marchionni, Dorian Belakebi, Marie Fabre, Alexandre Maria, Aurélie Du-Thanh

**Affiliations:** a Department of Dermatology, University Hospital of Montpellier, Montpellier, France; b Department of Internal Medicine, University Hospital of Montpellier, Montpellier, France

**Keywords:** drug-induced lupus, pembrolizumab, toxic epidermal necrolysis-like subacute cutaneous lupus erythematosus

## Introduction

Pembrolizumab, a widely used immune checkpoint inhibitor, is associated with various cutaneous adverse events. We report a particularly severe case of cutaneous immune-related adverse event (irAE), with a challenging diagnosis of epidermal necrolysis.

## Clinical case

**Fig 1:**
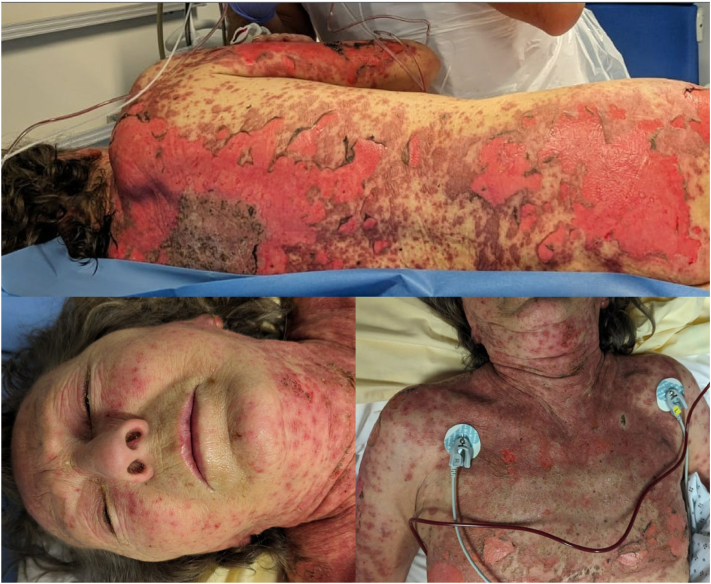
Epidermal detachment involving 40% of body surface area upon admission. Note the initial absence of cheilitis or mucosal involvement.

A 70-year-old woman with a history of metastatic lung adenocarcinoma was referred to the intensive care unit for diffuse erythema and epidermal detachment, affecting 40% of her body surface area. Her medical history included bipolar disorder, treated with haloperidol 10 mg daily for 10 years, without any other medication. She had previously completed 4 cycles of pembrolizumab combined with carboplatin and pemetrexed. She had reported an erythematous rash without mucosal involvement after the second cycle. The rash worsened after the third cycle, but she was not referred to a dermatologist. Patient was considered in remission after the fourth cycle, administered 2 weeks prior to her hospital admission. She had not undergone a recent computed tomography scan with contrast injection. Upon admission, the patient was apyretic. She presented with superficial flaccid bullae and large areas of epidermal detachment without mucosal involvement (Fig 1). She reported no unusual joint pain or swelling. Her psychiatric condition was stable. Two days after admission, she developed multiple, non-necrotic, well-demarcated erosive lesions of variable size involving the entire oral mucosa, with sparing of the tongue and a very mild cheilitis. Epidermal detachment reached 60% of her body surface area. No ocular or genital mucosal involvement was observed.

**Fig 2:**
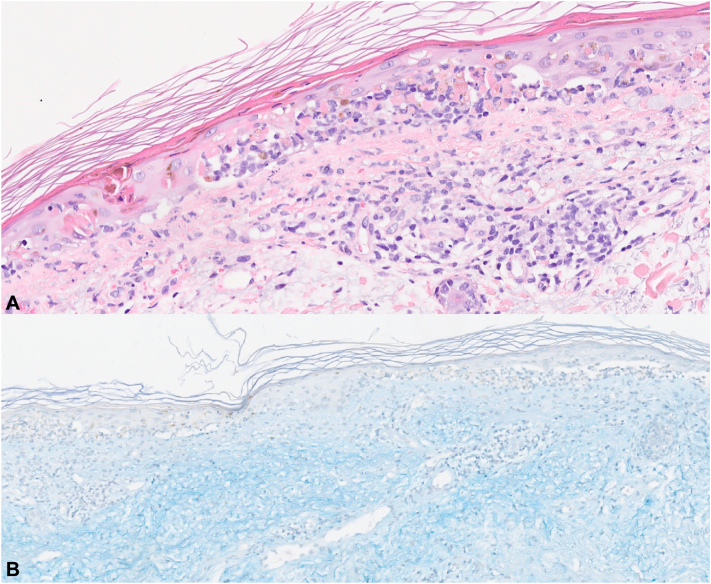
Histopathological examination of a skin biopsy: (**A**) hematoxylin and eosin staining at ×200 magnification and (**B**) Alcian blue staining at ×200 magnification.

Upon admission, laboratory tests showed normocytic anemia without hypereosinophilia, elevated inflammatory markers, and hypoalbuminemia. She had no renal dysfunction and no proteinuria. Antinuclear antibody (ANA) testing revealed a speckled pattern (1:640) along with positive anti-Sjögren's syndrome-related antigen A (SSA)/Ro (954 UA/mL) and anti-Sjögren's syndrome-related antigen B (SSB)/La (87 UA/mL). Perinuclear antineutrophil cytoplasmic antibodies staining was also positive (1:400). The levels of C3c and C4 were within normal range. Interestingly, ANA testing performed prior to her first cycle of pembrolizumab was already positive (ANA 1:1280), but did not lead to any specific follow-up. A skin biopsy from the patient’s dorsal region revealed vacuolar degeneration at the dermal-epidermal junction, keratinocyte necrosis, lymphocytic pericapillaritis, and mucin deposits in the reticular dermis (Fig 2). Direct immunofluorescence was negative.

The clinical presentation and evolution, histopathology, and abnormal autoantibody profiles led to a diagnosis of systemic lupus erythematosus (SLE) according to the 2019 European Alliance of Associations for Rheumatology (EULAR) criteria. The patient scored 19 points: leukopenia (+3), thrombocytopenia (+4), oral ulcers (+2), subacute cutaneous lupus (+4), and SLE-specific antibodies (+6). The initial clinical presentation mimicked toxic epidermal necrolysis (TEN), which ultimately led to the final diagnosis of drug-induced, TEN-like subacute cutaneous lupus erythematosus. According to the Bégaud algorithmic method, the imputability of pembrolizumab was evaluated as C2S2 for intrinsic causality and B3 for bibliographic evidence. This indicates a probable relationship, with compatible and characteristic clinical manifestations, and an established association in the literature.^1^

The patient was treated with 1 mg/kg oral prednisolone and 400 mg hydroxychloroquine daily. Her condition significantly improved within only 1 week. Prednisolone was gradually tapered and interrupted over 6 months. During this time, the eruption did not relapse, but postinflammatory patchy hyperpigmentation persisted. Pembrolizumab was discontinued due to this grade 4 adverse reaction, as defined by the Common Terminology Criteria for Adverse Events, along with other antineoplastic therapies. At the 3-month and 6-month follow-ups, the patient was still considered to be in cancer remission and remained under surveillance. A multidisciplinary team involving internists, oncologists, and dermatologists decided that, for this individual case, immunotherapy could be reintroduced in the absence of viable alternatives, using a different agent if possible. This would be done under continued hydroxychloroquine therapy, with the option to resume corticosteroids if needed, and close dermatologic monitoring.

## Discussion

Cutaneous irAEs occur in up to 34% of patients treated with programmed death (PD)-1 inhibitors.^2^ The PD-1/PD-L1 pathway maintains peripheral tolerance by inhibiting self-reactive T cells. Blocking this pathway can disrupt immune tolerance, potentially leading to autoimmune manifestations.^3^ SLE can be drug-induced, with therapeutic monoclonal antibodies (mAbs) being considered at low risk (approximately 0.1% of cases), although this effect is probably underestimated.^4^ When detected before the introduction of pembrolizumab, ANA positivity has been considered as a predictor of more severe irAEs.^5^ Moreover, the patient had reported an undefined skin rash right after the second cycle of pembrolizumab. These might have been the first SCLE lesions, but they were not investigated at the time.

According to a recent systematic review, nivolumab and pembrolizumab showed the highest rate of drug-induced subacute cutaneous lupus erythematosus (DI-SCLE) among mAb users (including anti-tumor necrosis factor). The authors defined mAb-induced SCLE as erythema multiforme-like, characterized by positive ANA and anti-Ro antibodies, photosensitivity (although less pronounced than nondrug-induced SCLE), and histological features such as interface dermatitis, lymphocytic infiltrate, necrotic/apoptotic keratinocytes, and mucin deposits in dermis. These manifestations occurred after a median of 3 drug doses. They identified 6 cases of DI-SCLE with pembrolizumab and 6 with nivolumab reported in the literature.^1^ They underlined that mAb-induced SCLE usually have a good prognosis, which is consistent with the full and rapid recovery of this patient despite an initial severe presentation.

The differential diagnosis between TEN/Stevens-Johnson Syndrome (TEN/SJS) and TEN-like DI-SCLE can be challenging because TEN/SJS can also be induced by immune checkpoint inhibitors.^6^ In our case, the absence of fever, the involvement of only one mucosal area, the presence of a marked interface dermatitis with a lymphocytic infiltrate, and mucin deposition in the dermis favored a DI-SCLE diagnosis. The absence of lupus band test in our case did not exclude the diagnosis because although very specific, its sensitivity in SCLE is low. Additionally, elevated anti-Ro antibodies are a relevant characteristic of TEN-like SCLE, drug-induced or not.^7^ These antibodies are rarely reported in TEN/SJS; however, they could be elevated in severe TEN/SJS because of a global acute immune response.^8^ Although the characteristics of the previous skin rash could not be defined *a posteriori* in our patient, the time course was consistent with delayed-onset cutaneous irAEs.^9^^,^^10^ Finally, the good and rapid response to corticosteroids and hydroxychloroquine further supports the diagnosis of TEN-like DI-SCLE (Table I).

**Table I:** Comparative features of toxic epidermal necrolysis and drug-induced, toxic epidermal necrolysis-like, subacute cutaneous lupus erythematosus

	Drug-induced, toxic epidermal necrolysis-like, subacute cutaneous lupus erythematosus	Stevens-Johnson syndrome/toxic epidermal necrolysis
Typical onset	Subacute or gradual	Acute and rapidly progressive
Skin lesions	Macules, erythema multiforme-like lesions, secondary epidermal detachment	Erythema multiforme-like lesions, purpuric lesions, diffuse erythema, bullae, secondary widespread epidermal detachment
Mucosal involvement	Frequent but usually limited	Severe and extensive (oral, ocular, genital mucosae)
Distribution	Initially on photoexposed areas	Widespread over >30% of the body surface area
Histopathology and direct immunofluorescence on skin biopsy	Interface dermatitis, apoptotic keratinocytes, mucin deposits, lupus band test	Necrotic keratinocytes, full-thickness epidermal necrosis, sparse inflammation
Immunologic findings	Usually ANA+, anti-Ro/SSA+	No specific antibodies∗
Systemic features	Arthritis and other systemic lupus symptoms	High fever, hemodynamic instability, multiorgan failure is possible
Prognosis and treatment	Rapidly favorable with corticosteroids or immunosuppressants and/or hydroxychloroquine	High mortality depending on the SCORTEN. Supportive care in intensive care unit, no standard treatment.

*ANA*, Antinuclear antibody; *SSA*, Sjögren's syndrome-related antigen A.

∗ Some authors report that ANA testing can be positive in toxic epidermal necrolysis.

This case highlights the critical need for close cooperation between oncologists and dermatologists in monitoring the rapidly increasing number of patients receiving PD-1 inhibitors, particularly those with preexisting autoimmune markers. Early identification of cutaneous irAEs, such as DI-SCLE, requires systematic referral to dermatologists to mitigate severity through appropriate treatments. This approach could ultimately improve patient outcomes and optimize the therapeutic efficacy of these antineoplastic agents.

## Conflicts of interest

None disclosed.
